# Neuronal and microglial regulators of cortical wiring: usual and novel guideposts

**DOI:** 10.3389/fnins.2015.00248

**Published:** 2015-07-17

**Authors:** Paola Squarzoni, Morgane S. Thion, Sonia Garel

**Affiliations:** Centre National de la Recherche Scientifique UMR8197, Ecole Normale Supérieure, Institut de Biologie, Institut National de la Santé et de la Recherche Médicale U1024Paris, France

**Keywords:** guidance molecules, glial cells, Cajal-Retzius cells, microglia, axon guidance

## Abstract

Neocortex functioning relies on the formation of complex networks that begin to be assembled during embryogenesis by highly stereotyped processes of cell migration and axonal navigation. The guidance of cells and axons is driven by extracellular cues, released along by final targets or intermediate targets located along specific pathways. In particular, guidepost cells, originally described in the grasshopper, are considered discrete, specialized cell populations located at crucial decision points along axonal trajectories that regulate tract formation. These cells are usually early-born, transient and act at short-range or via cell-cell contact. The vast majority of guidepost cells initially identified were glial cells, which play a role in the formation of important axonal tracts in the forebrain, such as the corpus callosum, anterior, and post-optic commissures as well as optic chiasm. In the last decades, tangential migrating neurons have also been found to participate in the guidance of principal axonal tracts in the forebrain. This is the case for several examples such as guideposts for the lateral olfactory tract (LOT), corridor cells, which open an internal path for thalamo-cortical axons and Cajal-Retzius cells that have been involved in the formation of the entorhino-hippocampal connections. More recently, microglia, the resident macrophages of the brain, were specifically observed at the crossroads of important neuronal migratory routes and axonal tract pathways during forebrain development. We furthermore found that microglia participate to the shaping of prenatal forebrain circuits, thereby opening novel perspectives on forebrain development and wiring. Here we will review the last findings on already known guidepost cell populations and will discuss the role of microglia as a potentially new class of atypical guidepost cells.

## Introduction

Functioning of the mammalian cerebral cortex relies on complex networks of axonal connections between neurons located in specific positions. The initial building of these exquisite circuits occurs during embryonic development and early post-natal days. During this “critical” period, neurons are first generated from spatially restricted proliferative niches and after or while reaching their final destination throughout active migration, extend oriented axons to form synaptic connections with their targets. Cellular migration is hence essential in the first part of brain wiring, because it allocates cells to specific positions and their subsequent settlement and differentiation, leading to the emergence of a functional system. In particular, cells can undertake radial or tangential migratory trajectories. During these processes, neurons can migrate locally or far away from their production sites as well as extend local axons or form long-range connections.

Because neurons are generated over a long-time period in the mammalian brain, neuronal migration and axonal navigation occur concomitantly during the constant process of brain development. How are these processes coordinated spatially and temporally to ensure the proper wiring of neural circuits? Over the last decades, this intriguing question has begun to receive answers in a developmental context in which cellular migration and axonal navigation take prominent places, namely the development of the embryonic mammalian forebrain (Borrell and Marin, 2006; Griveau et al., 2010; Villar-Cervino et al., 2013). In distinct regions of the embryonic mammalian forebrain, such as the dorsal cerebral cortex and the ventrally located subpallium, extensive events of radial and tangential migration reallocate neuronal populations and orient axonal navigation. For example, early-born neurons such as Cajal-Retzius cells spread out from different regions of origin to cover all the surface of the cortical primordium; inhibitory interneurons originate from the basal ganglia from which they tangentially migrate to populate the telencephalon; corticothalamic and thalamocortical axons traverse intermediate targets to reach their respective final targets. These events are fundamental to assemble cortical circuits and build the intricate circuitry essential for its functioning. Since defects in migratory processes during embryogenesis have been correlated with the onset of several neurologic and psychiatric diseases, it is crucial to decipher how they are regulated. Besides cells redistribution and morphogenesis, past and more recent studies showed that, throughout neuronal migration, an additional prominent event occurs during forebrain development that is the positioning of molecular cues, which instructs the trajectories of other migrating cells and growing axons. The cells that show these driving properties have different origins, but they share some common characteristics, for which they have been defined as guidepost cells. The purpose of this review is first to provide a definition of the usual concept of guidepost cells, giving an overview of already well-known examples. Moreover, we propose to extend the classical concept of guidepost cells, by speculating on recent findings concerning novel roles of microglia, the macrophages of the brain, in embryonic forebrain wiring.

## Toward a “modern” definition of guidepost cells

The concept of guidepost cells emerged from the studies on the developing limb bud of the grasshopper embryo (Borrell and Marin, 2006; Griveau et al., 2010; Kwon et al., 2011; Villar-Cervino et al., 2013). Bate and others described how pioneer projecting axons rely on some intermediate targets positioned along the future axonal path to follow a highly stereotyped pathway (Kwon et al., 2011). These intermediate targets consist of immature neuronal cells that show high affinity for the pioneer growth cones, and that are able, upon direct contact, to stabilize their filopodia and reorient the axonal growth cones on the pathway (Kwon et al., 2011). These important findings laid the foundation of the term “guidepost cells,” as located discontinuously along the future axonal trajectory providing short-range cues thereby precisely controlling axonal navigation.

Since these seminal studies, several other cases of guidepost cells have been reported in different organisms and developmental systems (Borrell and Marin, 2006; Griveau et al., 2010; Kwon et al., 2011; Villar-Cervino et al., 2013). To date, the vast majority of the identified guidepost cells in vertebrates belongs to the class of glial cells, such as the radial glia of the optic chiasma (Misson et al., 1988; Guillery et al., 1995; Marcus et al., 1995; Marcus and Mason, 1995; Wang et al., 1995), glial bridges of anterior and postoptic commissures (Silver et al., 1982; Pires-Neto et al., 1998; Barresi et al., 2005; Lent et al., 2005), floor plate cells (Tessier-Lavigne et al., 1988; Bovolenta and Dodd, 1990, 1991; Placzek et al., 1990; Campbell and Peterson, 1993; Kennedy et al., 1994; Serafini et al., 1994, 1996), boundary cap cells (Golding and Cohen, 1997; Fraher et al., 2007), glial cells of the corpus callosum (Silver et al., 1982, 1993; Silver and Ogawa, 1983; Shu and Richards, 2001; Shu et al., 2003a,b). More recently, some populations of tangential migrating neurons have also been discovered to play a guidepost role, with a consequent need to expand the conceptual definition (Sato et al., 1998; Lopez-Bendito et al., 2006; Niquille et al., 2009; Bielle et al., 2011; Hirata et al., 2012). Guidepost cells have been then defined as usually early born, discrete cell populations, with specialized functions that control and regulate axonal navigation, by being located at crucial decision points along the axonal trajectories. These cells can eventually extend an axon along the upcoming path of the tract and, in contrast to other long range-intermediate targets, guideposts act at short range or directly by cell-cell contact. They constitute decisive landmarks for guiding the axons along the correct pathways, which is a fundamental requirement for accurate circuitry assembly. The demonstration of their importance has been highlighted in different systems, by specific cell ablation experiments (Bentley and Caudy, 1983; Sretavan et al., 1995; Del Rio et al., 1997; Sato et al., 1998) and by the use of genetic mutants (Bovolenta and Dodd, 1990; Lopez-Bendito et al., 2006; Bielle et al., 2011), which resulted in aberrant pioneer axonal trajectories (Bielle et al., 2011), eventually with ectopic collateral branches formation (Bentley and Caudy, 1983; Bovolenta and Dodd, 1990) and in failure in axonal progression and in specific axonal innervation (Sretavan et al., 1995; Del Rio et al., 1997; Sato et al., 1998; Lopez-Bendito et al., 2006), respectively. The mechanism by which guidepost cells exert their function in guiding pioneer axonal tracts is throughout the secretion of guidance cues, which can act as attractive or repulsive signals. Both glial cells and tangential migrating guidepost cells have been found to express various families of guidance molecules or adhesion molecules, such as Slits (Erskine et al., 2000; Plump et al., 2002; Shu et al., 2003c), Robos (Bielle et al., 2011), Wnts (Keeble and Cooper, 2006), Neuregulin (Lopez-Bendito et al., 2006), Draxin (Islam et al., 2009), Ephrins (Williams et al., 2003; Mendes et al., 2006) or extracellular matrix proteins (Kuhn et al., 1995; Mandai et al., 2014). The correct positioning of the same migrating guidepost cells at the intermediate targets along the path is itself instructed by guidance cues (Kawasaki et al., 2006; Nomura et al., 2006; Ito et al., 2008; Bielle et al., 2011). Since these cells act mainly at short range or by cell-cell contact, their proper localisation is fundamental for the subsequent axonal tract development. This has been clearly shown in various guidance molecule mutant models in which altered positioning of guidepost cells led to consequent specific axonal pathfinding defects (Kawasaki et al., 2006; Bielle et al., 2011).

## Tangential migrating guidepost cells in the pathfinding of the lateral olfactory tract

**Figure 1:**
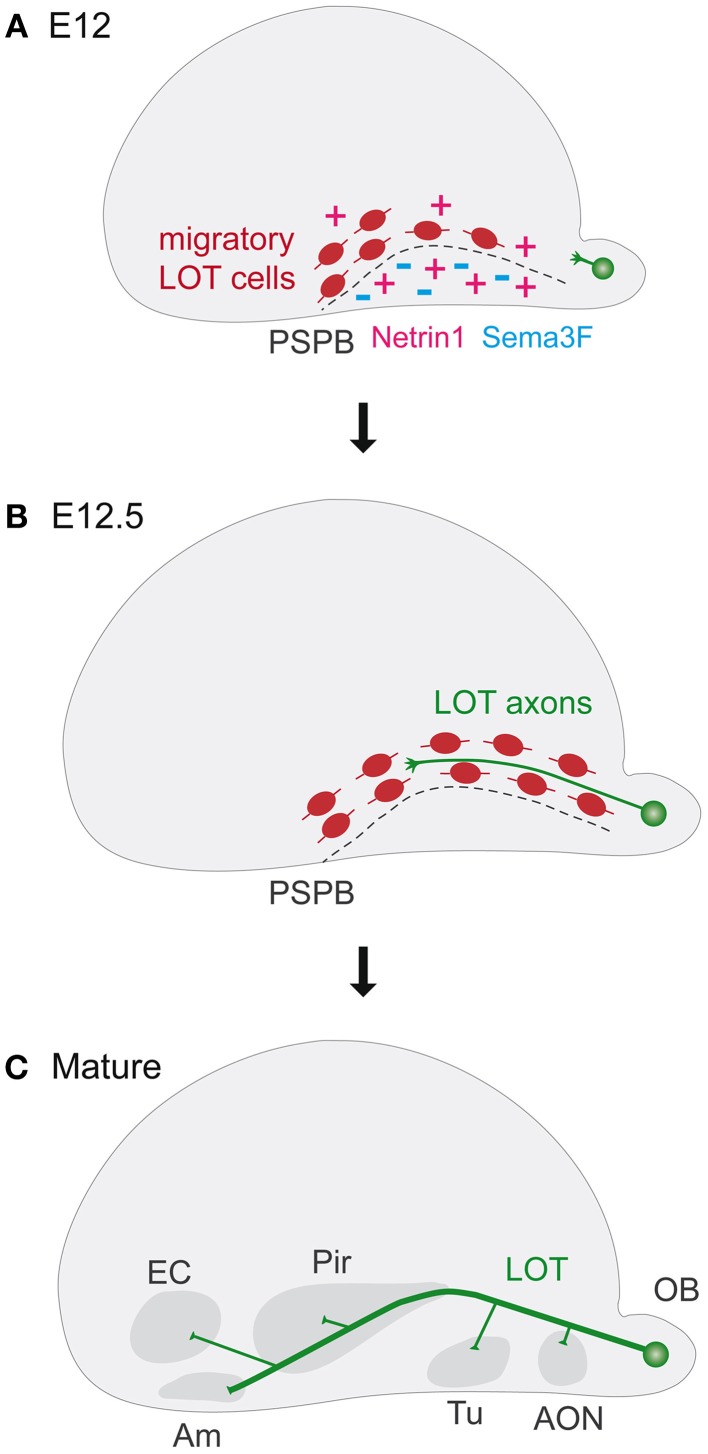
**Guidepost cells in lateral olfactory tract (LOT) development. (A–C)** The panels represent schematic lateral views of mouse embryonic cerebral vesicles. **(A)** Lot cells are amongst the first generated neurons in the brain. At E12 they are located along the pallial subpallial boundary (PSPB, dashes) before the arrival of LOT axons. Netrin1 attracts Lot cells toward the PSPB and Sema3F limits their migration. **(B)** At E12.5, LOT axons originating from the olfactory bulbs extend superficially in close contact with lot cells. **(C)** In the mature brain, the LOT contains axons projecting from the olfactory bulb to the anterior olfactory nucleus (AON), the olfactory tubercle (Tu), the piriform cortex (Pir), the entorhinal cortex (EC), and the amygdala (Am). Am, amygdala; AON, anterior olfactory nucleus; EC, entorhinal cortex; LOT, lateral olfactory tract; OB, olfactory bulb; Pir, piriform cortex; PSPB, pallial subpallial boundary; Tu, olfactory tubercle.

The Lateral Olfactory Tract (LOT) is the main efferent axonal bundle that conveys the olfactory information from the bulb to several higher olfactory centers in the brain, including the anterior olfactory nucleus, the olfactory tubercle, the piriform and entorhinal cortices and the amygdala (Borrell and Marin, 2006; Griveau et al., 2010; Villar-Cervino et al., 2013) (Figure 1). Indeed, sensory olfactory neurons, residing in the nasal cavities project onto tufted and mitral cells of the olfactory bulb, which in turn extend their axons into the LOT to reach cortical and associated regions. LOT pioneer axons initiate their outgrowth around embryonic day (E) 11.5, followed by the other mitral cells that collectively form the main axonal bundle around E13. Starting from E14.5, LOT axons extend superficial collaterals toward the olfactory cortices and into the other target regions.

Long-range guiding activities are involved in shaping the pathfinding of the LOT. Diffusible repulsive guidance proteins, such as Slit1 and Slit2 derived from the septum, regulate the lateral pathfinding of the mitral cell axons, throughout their receptors, Robo1 and Robo2 (Pini, 1993; Nguyen Ba-Charvet et al., 1999; Nguyen-Ba-Charvet et al., 2002; Fouquet et al., 2007). Some proteins of the Semaphorin class are involved in the growth of olfactory bulb axons (Sema3B) and repulsion of LOT axons (Sema3F) (De Castro et al., 1999; De Castro, 2009). Besides these diffusible long-range signals, it was shown that a peculiar population of cells supplies short-range permissive guidance activity in the formation of the LOT (Sugisaki et al., 1996). These “lot” cells have been identified by the expression of the lot1 antibody (Sato et al., 1998), recently shown to recognize the beta isoform of the metabotropic glutamate receptor subtype-1 (mGluR1) (Hirata et al., 2012). Lot cells are the first reported example of migrating neuronal guidepost cells involved in axonal pathfinding and are amongst the first generated neurons in the brain, around E9.5 and E11.5. They have been proposed to have a pallial origin and migrate toward the pallial subpallial boundary (PSB) (Tomioka et al., 2000). Once arrived in the PSB, they change their orientation and extend a long process toward the amygdala region (Kawasaki et al., 2006; Hirata et al., 2012). They correspond to previously identified cells horizontally disposed in the developing PSB (Derer et al., 1977) and their positioning occurs way before the arrival of LOT axons (Sato et al., 1998), for which they constitute a growing substrate. Around E12.5, the superficial growing of LOT axons displaces lot cells in the internal border of the path, where they are found subsequently in association with growing collateral axons (Hirata and Fujisawa, 1999). Although initially lot cells where considered as a distinct and unique cell population (Sato et al., 1998), these cells have been recently identified as a subset of Cajal-Retzius (CR) cells, a population of early born cortical neurons, since they share the expression of common molecular markers such as p 73 and Reelin (Dixit et al., 2014).

The role of CR-lot cells as guidepost for the developing LOT tract has been shown throughout toxic ablation experiments by the local use of a neuronal toxin, 6-hydoxydopamine, which provokes CR-lot cell death with consequent stall of mitral cell axons in strict proximity (Sato et al., 1998). This role is further highlighted by the analysis of mutant mice that affect this cell population such as Lhx2 (Saha et al., 2007) or Neurog1 and Neurog2 double mutants (Dixit et al., 2014). It has been proposed that lot cells form transient connections with LOT axons, as their final targets in the piriform cortex, amygdala and other higher olfactory centers are not yet mature (Sato et al., 1998; Hirata et al., 2012).

The importance of the proper positioning of CR-lot cells is thus highlighted by their guidepost function to orient LOT axons along their pathway. Still, how these cells act on axons and whether they are required for the progression, channeling or guidance of all axons remains largely to be characterized. By contrast, several guidance cues have been shown to play a role in the positioning of CR-lot cells in the ventral PSB. Netrin1 has been shown to act as an attractant cue for migrating CR-lot cells and participates, in part, to their ventral positioning (Kawasaki et al., 2006). However, in knockout mutant animals for *Netrin1* or its receptor *DCC*, only the location of the most ventral CR-lot cells resulted affected, associated with specific pathfinding defects on the ventral most LOT axons (Kawasaki et al., 2006). In double *Slit1; Slit2* mutants the LOT axonal tract is severely disrupted, with only few axons present in their correct positions. In this context, the proper positioning of CR-lot cells appears to be not drastically affected, thereby revealing that both long-range and local signals cooperate in LOT axonal pathfinding (Fouquet et al., 2007). Another important regulator of the ventral tangential migration of CR-lot cells is the molecule Sema3F that, by the interaction with its specific receptor neuropilin-2 (Nrp-2), confines CR-lot cells on the telencephalic surface (Ito et al., 2008). Sema3F, expressed in the subpallium and cortical plate, acts as a repellent signal, which prevents CR-lot cells to penetrate into deep brain regions, where some are ectopically found in case of *Sema3F* or *Nrp-2* invalidation (Ito et al., 2008). So far, there are not yet reported defects of LOT projections in *Nrp-2* mutants (Chen et al., 2000), raising the possibility that these guidepost cells may act locally. Furthermore, since many of these guidance cues can directly act on the axons, additional eventual effects of these genetic invalidations on the pathfinding of LOT axons deserve further analyses.

## Cajal-Retzius cells: Guideposts in the formation of entorhino-hippocampal projections

**Figure 2:**
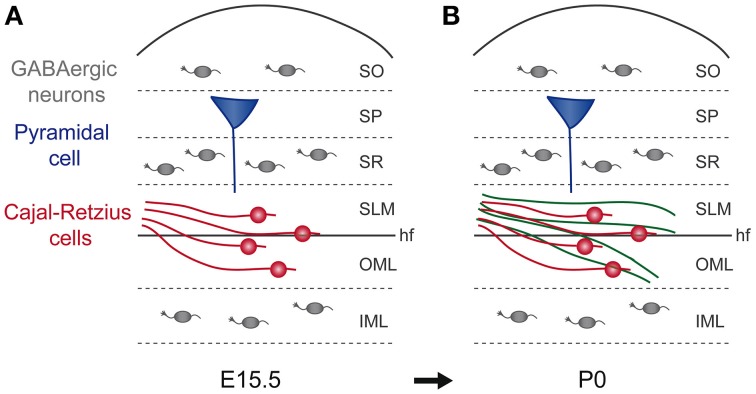
**Cajal-Retzius cells are guideposts for enthorhino-hippocampal axons. (A,B)** The panels represent schematic coronal sections of mouse hippocampi at E15.5 and P0. **(A)** At E15.5, Cajal–Retzius cells (red) are distributed in SLM and OML and GABAergic neurons (gray) are distributed in stratum oriens (SO), stratum radiatum (SR), and inner molecular layer (IML). **(B)** The major afferent excitatory projections in the hippocampus derive from pyramidal neurons in layers II and III of the entorhinal cortex. Between E16.5 and P0, entorhinal axons (green) invade specifically the SLM and OML in close association with Cajal–Retzius cells even if their future final target, the apical dendrites neurons of the pyramidal layer (SP, blue) develop later. hf, hippocampal fissure; IML, inner molecular layer; OML, outer molecular layer; SLM, stratum lacunosum-moleculare; SO, stratum oriens; SP, stratum pyramidale; SR, stratum radiatum.

Besides their emerging role in LOT axonal guidance, Cajal-Retzius cells, together with GABAergic interneurons, have been involved in the development of entorhino-hippocampal projections (Borrell and Marin, 2006; Griveau et al., 2010; Villar-Cervino et al., 2013). The major afferent excitatory projections in the hippocampus derive from pyramidal neurons in layers II and III of the entorhinal cortex. In particular, layer II pyramidal neurons form axonal connections with the dendrites of the granule cells of the outer molecular layer (OML) of the dentate gyrus (DG), whereas layer III neurons connect mainly with pyramidal cells in the stratum lacunosum-moleculare (SLM) in the cornu ammonis 1 and 3 (CA1 and CA3) (Borrell and Marin, 2006; Griveau et al., 2010; Villar-Cervino et al., 2013). Notably, during brain formation, the entorhinal axons already reach their final positions in the hippocampal regions, before the definitive development of their targets. Indeed, in mouse brain, entorhinal axons arrive in the hippocampus around E15, then they form arborisations in the SLM around E17 and are detected into the OML starting from the first postnatal day (Super and Soriano, 1994; Super et al., 1998; Deng and Elberger, 2001; Deng et al., 2006) (Figure 2). Therefore, even if hippocampal pyramidal neurons and granule cells are generated between E14 and E16, it is only around the second postnatal day that their apical dendrites start to be seen in the SLM, arising as final targets for entorhinal axons (Caviness, 1973; Soriano et al., 1986, 1989; Bayer and Altman, 1987; Super et al., 1998). This process of precise axonal addressing is regulated by Cajal-Retzius cells, which, as in LOT formation, have been reported to regulate axonal outgrowth. Cajal-Retzius (CR) cells are early born neurons, which are produced at E9-11 by focal pallial sources, including cortical hem, septum, PSB, and thalamic eminence (Grove et al., 1998; Meyer et al., 1999, 2002; Meyer and Wahle, 1999; Hevner et al., 2003; Takiguchi-Hayashi et al., 2004; Bielle et al., 2005; Cabrera-Socorro et al., 2007; Tissir et al., 2009; Ceci et al., 2010; Meyer, 2010; Gu et al., 2011; Martinez-Cerdeno and Noctor, 2014). CR cells migrate tangentially from their sources in the marginal zone of the cerebral cortex and rapidly cover the entire sheet. Their marginal localization and migration is regulated by CXCL12 produced by the meninges, which acts through CXCR4 and CXCR7 receptors (Borello and Pierani, 2010; Trousse et al., 2014). The marginal maintenance of CR cells also requires radial glia integrity, as revealed by analysis of β1 integrin conditional knockout (Kwon et al., 2011). In parallel, interactions between CR cells or with surrounding structures have been shown to control their random dispersion and distribution by Eph/ephrin-dependent contact repulsion or PlexinD1 signaling (Villar-Cervino et al., 2013; Bribian et al., 2014). Such migratory behaviors enable CR cells of different sources to preferentially cover cortical regions (Griveau et al., 2010; Gu et al., 2011). Functionally, CR cells have been shown to regulate cortical layering, neuronal and radial glia morphology, and cortical regionalisation, via the production of the secreted glycoprotein Reelin or additional membrane-bound or secreted factors (Borello and Pierani, 2010; Griveau et al., 2010; Gil-Sanz et al., 2013; Trousse et al., 2014). In the hippocampus, as the structure folds during development, CR cells localize in the future SLM and OML (Takiguchi-Hayashi et al., 2004; Bielle et al., 2005; Yoshida et al., 2006). Using electron microscopy, it has been shown that during embryogenesis pioneer entorhinal axons form transient synaptic contacts with Cajal-Retzius cells in SLM and OML, the future regions that will host pyramidal and granule cells apical dendrites (Super et al., 1998). Moreover, in organotypic culture experiments, this interaction has been shown to be fundamental for the growth of entorhinal axons in the hippocampus (Frotscher and Heimrich, 1993; Li et al., 1993; Frotscher et al., 1995; Del Rio et al., 1997). Indeed, eliminating CR cells from cultured slices with the local toxin 6-hydoxydopamine avoided entorhinal axonal growing in the hippocampus (Del Rio et al., 1997). These experiments therefore constituted a strong evidence of the role as placeholders for CR cells in the formation of entorhino-hippocampal connections (Forster et al., 1998). About the molecular cues that could be involved in entorhinal axon guidance by CR cells, the most obvious candidate could be the glycoprotein Reelin, of which CR cells constitute the main source (D'arcangelo et al., 1995, 1997; Hirotsune et al., 1995; Ogawa et al., 1995; Tissir and Goffinet, 2003). It has been previously shown that Reelin controls cortical layering, organization and the orientation of radially migrating neurons (Borello and Pierani, 2010; Frotscher, 2010; Griveau et al., 2010; Martinez-Cerdeno and Noctor, 2014). Nevertheless, antibody-mediated blocking of Reelin in *ex vivo* co-cultures of hippocampal slices and entorhinal tissue, does not lead to dramatic defects in entorhinal axonal pathfinding. However, fewer entorhinal fibers reach the hippocampal layers, developing shorter axonal branches. These findings have been confirmed *in vivo* in *reeler* mice, a natural Reelin mutant, which presents severe defects in cortical lamination. Similarly to co-cultures experiments, the absence of Reelin had no dramatic effects about entorhinal axonal ingrowth or targeting, but entorhino-hippocampal axonal terminations appear thinner than in control animals. Moreover, these defects are transient, since then in *reeler* adult mice a normal branching density is observable (Frotscher and Heimrich, 1993; Li et al., 1993; Frotscher et al., 1995; Del Rio et al., 1997; Borrell et al., 1999; Deller et al., 1999). Altogether, these results confirm the important role of CR as guidepost cells in entorhino-hippocampal innervation and reveal reelin as an important factor for branching, collateral formation and synaptogenesis of entorhinal axons. However, they leave still an open question about additional molecular cues involved in the pathfinding of entorhinal axons in the hippocampus.

## En route to the cortex: Guidepost cells open a path for thalamocortical connections

Mammalian neocortex forms connections with the rest of the brain via the internal capsule, which includes bundles of corticofugal efferent axons and reciprocal afferent thalamocortical projections, which convey sensory and motor information to the neocortex. In the context of axonal pathfinding, the development of the internal capsule has been extensively studied (Molnar et al., 2012; Garel and Lopez-Bendito, 2014). Indeed, this system has a major physiological relevance, but also allows a variety of experimental approaches, due to its important size and extension in the developing brain.

**Figure 3:**
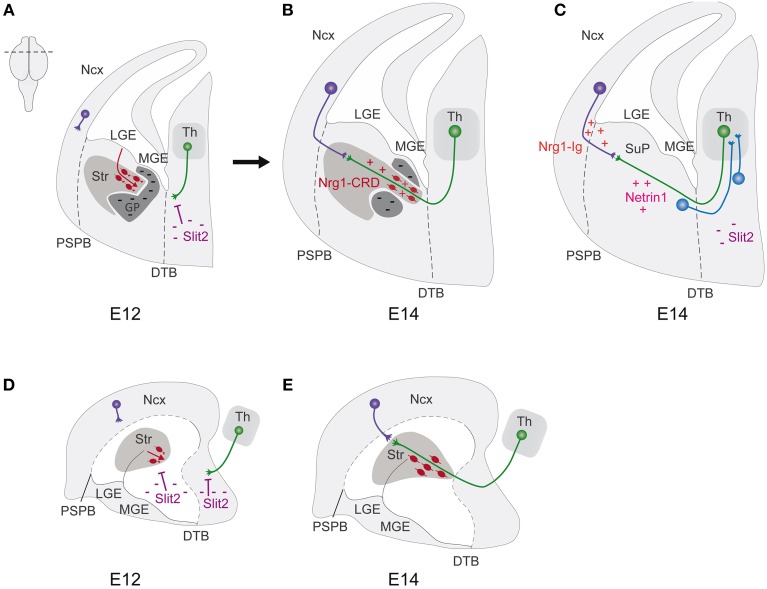
**Corridor neurons shape the internal pathfinding of thalamocortical axons. (A–C)** Schematic representation of hemicoronal sections of mouse embryonic telencephalons. **(D,E)** Schematic representation of median sagittal views of mouse embryonic telencephalons. **(A,D)** At E12, tangentially migrating LGE-cells (red), repelled by Slit2, form in the MGE a permissive corridor for thalamocortical axons. Thalamocortical axons (green), repelled by hypothalamic Slit2, turn to enter the MGE. **(B,E)** At E14, pioneer thalamocortical axons grow through the permissive corridor (red cells) and the Str, where they encounter the reciprocal pioneer corticothalamic axons (purple). Guidance cues including Slits, Netrin1, Nrg1-IG, Ng1-CRD present along the pathway, orient the axons. **(C)** Thalamocortical axons (green) extend from the dorsal thalamus (Th) toward the neocortex (Ncx), crossing the diencephalic-telencephalic boundary (DTB, black dashes), and enter the subpallium (SuP) at the level of the internal capsule. At E14, thalamocortical axons reach the pallial subpallial boundary (PSPB, black dashes), where they encounter the reciprocal pioneer corticothalamic axons (purple). Thalamocortical axon guidance is regulated by different cellular and molecular actors: prethalamus (blue) and SuP cells sending an axon to the Th; the repellent Slit2 in the hypothalamus, the attractant Netrin1 in the SuP, Nrg1-Ig in the neocortex. DTB, diencephalic-telencephalic boundary; GP, globus pallidus; LGE, lateral ganglionic eminence; MGE, medial ganglionic eminence; Ncx, neocortex; PSPB, pallial subpallial boundary; Str, striatum; SuP, subpallium; Th, dorsal thalamus.

During the years, many findings had contributed to elucidate the routes and the molecular mechanisms that shape thalamocortical and corticofugal connection paths. In mouse development, these important axonal systems start to form during early/mid gestation. Thalamocortical axons (TCAs) originate from neurons located in the thalamus, grouped in distinct nuclei, showing a topographic organization that corresponds to the spatial innervation of different cortical areas (Molnar et al., 2012; Garel and Lopez-Bendito, 2014). From E12 to E15, TCAs extend ventrally, crossing the prethalamus, and traverse the diencephalic/telencephalic boundary, entering the subpallium at the level of the internal capsule. At E14, early TCAs reach the PSB, where they encounter the reciprocal pioneer corticothalamic axons (CTAs). Subsequently, from E14.5 to E18.5, TCAs form transient connections with subplate cells residing in their respective target cortical areas. After this waiting period, TCAs send collaterals into the cortical plate and finally establish thalamocortical connections (Figure 3). Meanwhile, corticofugal axons grow along the same path of TCAs and split in CTAs and in corticosubcerebral axons that proceed toward other subcortical regions. This reciprocal wiring has been shown to be tightly controlled, in part by guidepost cells, transient axonal populations and several structures that have been shown to act as milestones along the path.

Chronologically, pioneer cortical subplate neurons have been firstly proposed as guidepost cells in regulating the entering and progression of TCAs into the cortical plate (Garel and Lopez-Bendito, 2014; Hoerder-Suabedissen and Molnar, 2015). These observations have been strongly supported by several experimental evidences. To date, in the visual cortex, subplate neurons ablation avoids the entering of the corresponding thalamic geniculocortical axons (Ghosh et al., 1990; Ghosh and Shatz, 1993). In mutant mice, such as *reeler, p35*^−∕−^ and *cdk5*^−∕−^ that present subplate cells in the marginal zone, due to severe defects in preplate splitting, TCAs form abnormal projections to connect with the ectopic subplate in the marginal zone. Since the discovery of subplate cells, other groups of cells have been found to exert a role of guidepost for TCAs. In mouse, early born cells, named perireticular cells, have been identified in the future path of the internal capsule, which at E12.5 send a cellular process to the thalamus. The hypothesized role of these cells is to provide a cellular scaffold for the future TCAs and CTAs, which is consistent with several experimental evidences (Mitrofanis, 1992, 1994; Mitrofanis and Baker, 1993; Mitrofanis and Guillery, 1993; Adams and Baker, 1995; Metin and Godement, 1996; Molnar et al., 1998b; Braisted et al., 1999; Molnar and Cordery, 1999). In effect, different defects in TCA pathfinding have been reported in mutant mice presenting absence, reduction or displacement of perireticular cells (Tuttle et al., 1999; Bishop et al., 2000, 2003; Lopez-Bendito et al., 2002; Lakhina et al., 2007). Unfortunately, the current absence of specific molecular markers and the wide distribution of these cells experimentally limit the investigation about their origin and function.

More recently, another population of guidepost cells has been observed in the subpallium, which comprises the lateral and medial ganglionic eminences (LGE and MGE). These cells are GABAergic LGE derived-neurons that tangentially migrate in the MGE, forming a permissive corridor, in an otherwise not permissive territory, for the growth of TCAs along an internal path toward the cortex (Lopez-Bendito et al., 2006). Because of their function, they have been named “corridor” cells; they are located in the MGE, in which they migrate from E11.5 to E14, but express LGE molecular markers, such as Islet1, *Ebf1* and *Meis2*. By gain-of-function experiments in cultured organotypic embryonic brain slices and by the use of full or conditional mutant mice of ErbB4 and Neuregulin1 respectively, it has been revealed that corridor cells, via the expression of Neuregulin1, provide a permissive corridor for TCAs, which express the corresponding ErbB4 receptor (Lopez-Bendito et al., 2006). How are corridor cells positioned? A ventral repulsive activity from the subpallium, mediated by Slit2 and Robo1 and Robo2 respective receptors, has been shown to limit, *in vitro, ex vivo*, and *in vivo*, the ventral tangential migration of corridor cells, playing a role in the formation of corridor shape. Indeed, *Slit2* inactivation leads to abnormal ventral migration of corridor cells, with aberrant corridor shaping and consequent defects on TCAs pathfinding (Bielle et al., 2011). These findings constitute a starting point for further investigations on the origin, specification and fate of corridor cells.

In addition to guidepost cells, several important structures and molecules present in the subpallium or prethalamus have been shown to play critical roles in the guidance of both TCAs and reciprocal CTAs (Metin and Godement, 1996; Braisted et al., 1999; Garel et al., 1999; Sussel et al., 1999; Marin et al., 2002; Marin and Rubenstein, 2002; Yun et al., 2003). For instance, knockout mutation analyses, have highlighted the importance of classical guidance cues and their receptors, such as Slit1, Slit2, Robo1, Robo2, Netrin1, and Sema6A, for the local regulation of TCAs guidance (Braisted et al., 2000, 2009; Leighton et al., 2001; Bagri et al., 2002; Bonnin et al., 2007; Lopez-Bendito et al., 2007; Powell et al., 2008; Little et al., 2009). Other important regulators are the members of the protocadherin family, which have been shown to be important for both TCAs and CTAs progression into the subpallium (Wang et al., 2002, 2006; Tissir et al., 2005; Uemura et al., 2007; Zhou et al., 2008, 2009; Qu et al., 2014). For instance, inactivation of OL-protocadherin was shown to impair the subpallial crossing of TCA associated with defects in striatal axonal outgrowth (Uemura et al., 2007). In addition, mutant mice with constitutive or specific inactivation of *Celsr3* in the prethalamus and subpallium present similar impairments, which consist in the stall of TCAs in the ventral subpallium, across the diencephalic/telencephalic boundary, and in CTAs arrest in the proximal part of the LGE, suggesting a possible cooperation of these two factors in the process (Tissir et al., 2005; Zhou et al., 2008, 2009; Qu et al., 2014). Strikingly, these phenotypes are almost phenocopied by deletion of *Frizzled3*, which is associated with *Celsr* signaling pathway (Wang et al., 2002, 2006). Last, but not least, the expression of the transmembrane protein Linx is required on subplate cells, subpallium and prethalamaus guideposts, for the progression of TCAs and CTAs revealing a role for this molecule in axon/axon interactions and potentially guideposts/axons interactions (Mandai et al., 2014). More generally, it will be essential to precise which of the aforementioned guidance cues regulates the positioning and/or function of guidepost cells located along the internal capsule path.

In addition to delineating an internal trajectory for TCAs, guidepost neurons have been shown to play additional roles in thalamo-cortical wiring. First, there is now solid evidence that TCAs and CTAs interact to form reciprocal connections, as proposed by the handshake hypothesis (Blakemore and Molnar, 1990; Molnar and Blakemore, 1991, 1995; Chen et al., 2012; Molnar et al., 2012; Deck et al., 2013; Garel and Lopez-Bendito, 2014). As such, guideposts that shape TCAs path have an indirect impact on the guidance of reciprocal CTAs. Second, while TCAs originating from principal thalamic nuclei all grow internally in the corridor, they adopt distinct rostrocaudal positions in the capsule, depending on their nucleus of origin and their cortical target (Molnar et al., 1998a, 2012; Garel and Lopez-Bendito, 2014). This topographic ordering has been shown to depend on local subpallial positional information (Dufour et al., 2003; Bonnin et al., 2007; Wright et al., 2007; Powell et al., 2008; Bielle et al., 2011; Demyanenko et al., 2011a,b; Lokmane et al., 2013). Indeed, guidance factors such as Slit1 and Netrin1 (and their combinatorial activity), Sema3A, ephrinAs, as well as L1, CHL1 participate to the topographic ordering of TCAs deriving from different thalamic nuclei, with a dramatic impact on their final cortical addressing (Bonnin et al., 2007; Wright et al., 2007; Powell et al., 2008; Bielle et al., 2011; Demyanenko et al., 2011a,b; Lokmane et al., 2013). Remarkably, positional information has been shown to be present already in the corridor, as TCAs enter the subpallium (Bielle et al., 2011). Accordingly, the aforementioned guidance factors are present in the corridor, especially Slit1, supporting the idea that they act as TCAs grow internally (Bielle et al., 2011). Importantly, altering the ordering of TCAs in the subpallium by genetic manipulation has been shown to impair the fine topography of TCAs in the somatosensory cortex (Lokmane et al., 2013; Lokmane and Garel, 2014). These experiments reveal that in addition to cortical signals, intermediate ordering of axons, in part by corridor cells, is important for fine-grained topography of TCAs. Together, such recent studies highlight additional roles of corridor internal guideposts in reciprocal and topographical wiring.

## Microglia cells: Novel unusual guidepost cells?

Microglia are the resident macrophages of the brain, which control brain homeostasis in physiologic conditions and constitute the first line of defense in case of diseases and against pathological threats. Initially described by Del Rio-Hortega (1932), the physiological functions and the origin of these cells have been remained controversial for a long time. Until recently, most studies focused on the roles of microglia in brain damage and diseases and in their participation to neuro-inflammatory processes via the release of neurotrophic and pro-inflammatory factors as well as the ability to perform phagocytosis. Over last decade, several landmark studies have revealed that, using conserved cellular mechanisms, microglia contribute to normal brain functions. Indeed, microglia have been shown to modulate synaptic transmission, to regulate synaptic formation and elimination, and to shape postnatal and embryonic brain circuits as reviewed in Paolicelli and Gross (2011), Schafer et al. (2013), Bilimoria and Stevens (2014), Katsumoto et al. (2014), Paolicelli et al. (2014), Salter and Beggs (2014) and Casano and Peri (2015). Below, we will focus on specific features of microglia during early brain wiring that bear similarities with those of guideposts cells, such as their capacity to act at short range, their early origin and focal positioning as well as the production of several molecular factors by which they can interact with and condition their surrounding neural environment.

### Microglia survey and interact with their local environment

In the last decade, technological advancements such as two-photon laser scanning microscopy, allowed the observation of microglia behavior *in vivo*, in normal conditions. Ramified microglia, initially thought to be in a resting state in opposition to the activated, amoeboid morphology observed following brain injury, were found to be extremely active in surveying their environment. Indeed, very frequent extensions and retractions of microglia ramifications were observed in contact with neighboring neuronal cells, astrocytes and blood vessels; furthermore their extensions were increased by changes in both neuronal activity, blood vessel lesions and ATP variation levels *in vivo* (Davalos et al., 2005; Nimmerjahn et al., 2005). Processes of “resting microglia” were found to interact with synapses in somatosensory and visual neocortex, forming direct appositions with different synaptic elements. In particular, morphological changes of microglia processes, with the appearance of phagocytic structures and modifications of synaptic apposition frequencies were shown to be modulated by variation in visual experience (Wake et al., 2009; Tremblay et al., 2010). Following similar lines, microglia were found to modulate neuronal activity (Li et al., 2012; Pascual et al., 2012). For instance, in zebrafish larvae optic tectum, microglia were shown to contact highly activated neurons for longer time, correlating with a subsequent decreased neuronal activity (Li et al., 2012). Conversely, microglia activation *in vitro* by LPS stimulation was reported to indirectly increase the frequency of spontaneous synaptic AMPAergic post-synaptic currents in hippocampal neurons (Pascual et al., 2012). Such findings have fundamentally changed our conception of microglia by revealing that these cells exert the capacity to act at short-range on their surrounding neural environment. Since then, besides their immune-defensives functions, these cells have started to be considered as active modulators during healthy brain development and maturation as well as actors of pathologic brain wiring and functioning.

### Early origin of microglia

Originally thought to arise form peripheral bone marrow derived-macrophages that invade the brain after birth, microglia have been show, throughout series of fate-mapping experiments, to originate from yolk sac myeloid progenitors and to be dependent on Pu.1, Irf8 (Kierdorf et al., 2013) and colony-stimulating factor 1 receptor (CSF1R) (Ginhoux et al., 2010, 2013; Erblich et al., 2011; Schulz et al., 2012; Gomez Perdiguero et al., 2013; Kierdorf et al., 2013; Hoeffel et al., 2015). In mice, yolk sac derived-microglia precursors migrate into the neural folds during embryogenesis and, by *in situ* proliferation, generate microglia that populate the adult brain. Under normal conditions, microglia comprise resident cells since the infiltration of peripheral monocytes or macrophages into the CNS is limited by the blood-brain barrier (Mildner et al., 2007; Ginhoux et al., 2010; Schulz et al., 2012; Gomez Perdiguero et al., 2013). Thus, microglia enter the brain from early prenatal stages and form an autonomous, self-sustained population. Remarkably, colonization of embryonic brain tissues by microglia appears to be a highly conserved process across vertebrate species (Perry et al., 1985; Ashwell, 1991; Cuadros and Navascues, 2001; Herbomel et al., 2001; Verney et al., 2010; Schlegelmilch et al., 2011; Swinnen et al., 2013), suggesting that embryological “seeding” of the microglial population may be also conserved.

How is the number or density of microglia regulated? Different embryonic or postnatal methods have been reported for the ablation of microglia *in vivo* or in cultured brain slices (Duffield et al., 2005; Heppner et al., 2005; Varvel et al., 2012; Ueno et al., 2013; Elmore et al., 2014; Squarzoni et al., 2014). Among those methods, pharmacologic depletion models acting on CSF1R signaling, revealed that after birth, a complete microglia repopulation occurs in a 1-week time window (Elmore et al., 2014; Squarzoni et al., 2014). These results show that there is a homeostatic control over the microglial population and raise the questions of the underlying mechanisms. While the origin of these repopulating cells is still debated, it has been shown for the adult repopulation that a local brain pool of nestin-positive cells differentiates into microglia thereby restoring their usual number (Elmore et al., 2014). Collectively, these essential findings match some forward-looking theories formulated by del Rio Hortega, which postulated that microglia enter the brain during embryogenesis (Del Rio-Hortega, 1932); at the same time, they highlight how the constant presence of microglia within the brain is tightly regulated.

### Microglia in defining the number of neurons: Neurogenesis and survival

Microglia have been recently shown to take part to several important events which contribute to shaping of neural circuits, including neurogenesis, neuronal survival, synaptic remodeling and maturation. The role in neurogenesis and survival has been examined in both the adult niche and the developing brain. For instance, in adult murine hippocampus, unchallenged microglia regulate by phagocytosis the number of immature neurons maintained in the subventricular zone, one of the few sites of postnatal neurogenesis (Sierra et al., 2010, 2013). In macaque and rat neocortex, alteration of microglia activity by maternal immune activation through LPS, Doxycycline treatment or microglia elimination by Liposomal clodronate exposition, significantly affects the number of neuronal precursors in the embryonic and postnatal brain (Cunningham et al., 2013).

In parallel, microglia have been also reported to regulate neuronal number by active induction of apoptosis, or oppositely to contribute to neuronal survival, in different regions of the brain. For instance, early postnatal apoptosis in the cerebellar Purkinje cell (PC) population was shown to be induced by superoxide ions generated from microglial respiratory bursts (Marin-Teva et al., 2004). These results provided support to previous studies showing that the depletion of microglia in brain culture slices *in vitro* resulted in increased PC survival (Van Rooijen et al., 1997). Likewise, in perinatal mouse hippocampus, microglia was found to enhance hippocampal neuronal apoptosis by the CD11b/DAP12 integrin signaling-dependent production of reactive oxygen species (Wakselman et al., 2008). Conversely, microglia have been shown to actively sustain postnatal cell survival of layer V cortical neurons in mouse by the production of the trophic factor IGF1 (Ueno et al., 2013). Indeed, postnatal microglia inactivation by minocycline, microglia temporal elimination in CD11b-DTR transgenic models, as well as the use of IGF1R inhibitors and *igf1* siRNA, resulted in increased cell death of layer V cortical neurons (Ueno et al., 2013). Altogether, these findings show that microglia regulate the number of neurons produced and maintained in the brain, through a balanced activity on progenitors, immature neurons and maturing neurons.

### Microglia shape the postnatal brain: Synapse formation and synapse pruning

Besides their roles on neurogenesis and neuronal cell homeostasis, microglia have been found to contribute to synaptogenesis, synaptic remodeling and brain maturation. Similarly to IGF1 for layer V cortical neurons, the production of the neurotrophin BDNF by microglia has been shown to promote synapse formation via signaling to its cognate receptor TrkB. Remarkably, specific microglia or microglia-BDNF depletion, using *CX3CR1*^*CreER*^ mice, both lead to deficits in multiple learning tasks and learning-induced synaptic remodeling (Parkhurst et al., 2013). These major findings highlight the ability of microglial cells to impact on the building and homeostasis of neural circuits throughout the very local active production of secreted factors.

In addition, microglia were shown to play an active role in postnatal synaptic pruning, contributing to the shaping and maturation of the brain, by their close spatial and temporal contact with synapses (Paolicelli et al., 2011; Schafer et al., 2012, 2013; Kettenmann et al., 2013). In particular, this has been directly observed in the retinogeniculate system, where surrounding microglia participate to the activity-dependent synaptic remodeling, eliminating the weaker presynaptic connections through a C3/CR3 complement-dependent mechanism (Schafer et al., 2012, 2013). Similarly, in the hippocampus microglia were found to contribute to synaptic refinement. Specifically, the CX3CR1/fractalkine signaling pathway plays a central role in microglia/synapses communication, since *Cx3cr1*^−∕−^ mice show temporal reduction of hippocampal microglia number, leading to a deficit in synaptic pruning. Consistently, with early defects in synaptic communication, these mice were shown to exhibit reduced functional brain connectivity, together with social interaction and behavioral deficits (Paolicelli et al., 2011; Zhan et al., 2014). In the somatosensory neocortex, reduced density of microglia cells of *Cx3cr1*^−∕−^ mice due to a delay in recruitment of these cells, has been shown to impact on the maturation of thalamocortical synapses (Hoshiko et al., 2012). Thus, the density of these cells, their proper functioning, as well as their capacity to specifically perform local phagocytosis or production of secreted factors, constitutes an important factor for sculpting postnatal brain circuits.

### Microglia in the embryonic and perinatal brain: The importance of spatial and temporal positioning

What about a role of microglia during embryogenesis? In contrast to their later homogeneous distribution in the adult brain, embryonic, and perinatal microglia show an uneven distribution in different species (Ashwell, 1991; Verney et al., 2010; Arnoux et al., 2013; Cunningham et al., 2013; Swinnen et al., 2013; Squarzoni et al., 2014). In particular, in the mouse, round or more ramified microglia have been observed in different focal hotspots, which are not particularly related to apoptosis, whereas some zones, such as the cortical plate is largely devoid of microglial cells (Ashwell, 1991; Verney et al., 2010; Cunningham et al., 2013; Swinnen et al., 2013; Squarzoni et al., 2014). More in depth analyses revealed that microglia accumulations correspond to important decision landmarks in axonal paths or cellular migratory routes. In particular, discrete groups of microglia associate with the corpus callosum, the external capsule or establish a contact with incoming dopaminergic axons in the ventral telencephalon. Specific associations with progenitor zones have also been observed in mice and other mammals, potentially regulated by chemokine production by these progenitors (Cunningham et al., 2013; Arno et al., 2014).

**Figure 4:**
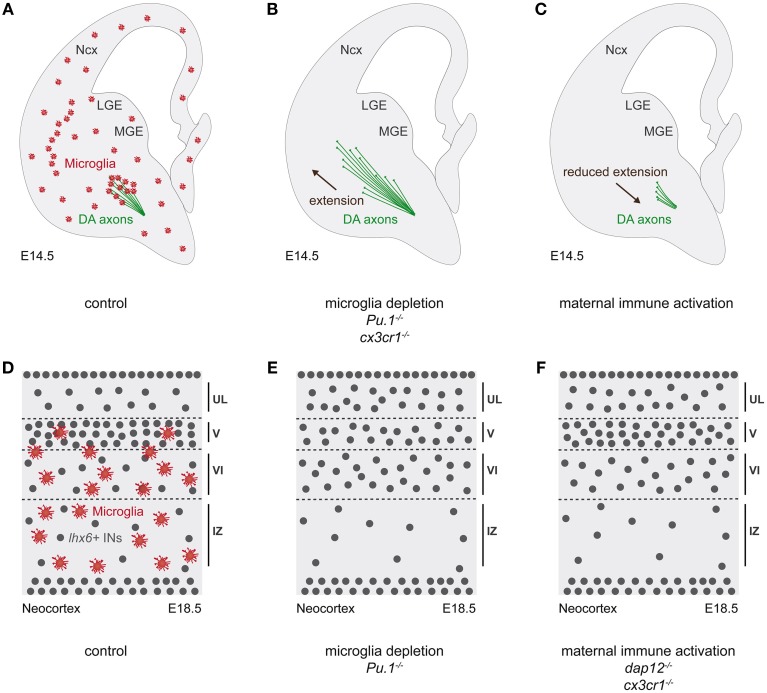
**Microglia in the outgrowth of dopaminergic axons and the positioning of cortical interneurons. (A–C)** Schematic representations of coronal hemisections of E14.5 mouse embryonic brain. **(D–F)** Schematic representations of E18.5 coronal sections through the somatosensory neocortex of mouse embryonic brain. **(A)** At E14.5, microglia (red) establish a contact with dopaminergic axons (green) entering in the ventral telencephalon. **(B)** Pharmacologic or genetic cellular ablation (*Pu1*^−∕−^), as well as functional impairment (*Cx3cr1*^−∕−^) of microglia promote dopaminergic axonal outgrowth in the striatum. **(C)** Conversely, microglia functional alteration by maternal immune activation (MIA) leads to reduced dopaminergic axonal outgrowth. **(D)** At E18.5, microglia localize in the deeper layers of the cortical plate, with *lhx6*-expressing interneurons (gray dots) being concentrated in layer V. **(E,F)** In absence of microglia (pharmacological depletion or *Pu1*^−∕−^) pharmacological immune activation (MIA) or genetic functional alteration of microglia (*Dap12*^−∕−^; *Cx3cr1*^−∕−^), *lhx6*-expressing interneurons prematurely entered the cortical plate, followed by an altered laminar distribution. DA, dopaminergic axons; INs, interneurons; IZ, intermediate zone; LGE, lateral ganglionic eminence; MGE, medial ganglionic eminence; Ncx, neocortex; UL, upper layers.

Pharmacologic or genetic ablations of microglia have been used to probe the roles of these cells during embryonic brain wiring (Figure 4). Together with maternal immune activation (MIA) and genetic microglial impairment (*Cx3cr1*^−∕−^), these studies showed that microglia regulate the outgrowth of dopaminergic axons, thereby revealing the importance of the precise spatial-temporal microglia localisation (Squarzoni et al., 2014). In addition, microglia contribute to the development of the Corpus Callosum (CC), the largest commissural structure between the cerebral hemispheres (Pont-Lezica et al., 2014). Indeed, genetic functional impairment of microglia (*Dap12*^−∕−^) or developmental functional alteration by MIA, down-regulate the expression of genes related to neuritogenesis in microglia, with a consequent impairment on the CC fasciculation in these mouse models. A similar CC fasciculation phenotype has been equally observed in the genetic model of microglia ablation, *Pu*·*1*^−∕−^ (Pont-Lezica et al., 2014). Together these studies suggest that the spatial and temporal positioning of embryonic microglia modulates the development of specific and important axonal tracts. The underlying cellular and molecular mechanisms still remain to be deciphered.

In addition, microglia, which show a timely invasion of the cortical plate (CP) (Cunningham et al., 2013; Swinnen et al., 2013; Squarzoni et al., 2014) were found to regulate the assembly of cortical circuits. Cortical circuits are formed by an intricate network of a majority of excitatory neurons and a minority of functionally important inhibitory interneurons (Marin and Rubenstein, 2003; Sur and Rubenstein, 2005; Batista-Brito and Fishell, 2009; Cossart, 2011; Fishell and Rudy, 2011; Rico and Marin, 2011; Rubenstein, 2011; Marin and Muller, 2014). Indeed, various classes of interneurons shape the network output and interneuron dysfunction as well as defects in the excitation/inhibition balance have been associated with several neurodevelopmental disorders such as Autism Spectrum Disorders (ASD) or Schizophrenia. As aforementioned, microglia regulate the number of neuronal precursors in the subventricular zones of the neocortex (Cunningham et al., 2013) and are firstly excluded from the CP, which they invade after E16.5, remaining initially confined to the deeper layers (Swinnen et al., 2013; Squarzoni et al., 2014). Absence, immune activation or genetic impairment of microglia were found to impact on the laminar distribution of a specific population of interneurons that express the transcription factor Lhx6. Indeed, in absence of microglia (pharmacological depletion; *Pu*·*1*^−∕−^), or in case of pharmacological (MIA) or genetic functional alteration (*Dap12*^−∕−^; *Cx3cr1*^−∕−^), a premature entry of *lhx6*-expressing interneurons in the CP was observed, followed by an altered laminar distribution, with long lasting postnatal effects on a subset of *lhx6*-expressing interneurons, the fast-spiking parvalbumin-positive interneurons (Squarzoni et al., 2014). These specific interneurons have been shown to play a major role in cortical networks as well as to be impaired in ASD and Schizophrenia (Penagarikano et al., 2011; Marin, 2012; Meechan et al., 2012). While these results reveal a surprising role of microglia in cortical circuits assembly, as well as a potential involvement in the etiology of neuropsychiatric diseases, they raise the question of the underlying mechanisms. Besides the requirement of Dap12 and Cx3cr1 signaling, the processes involved deserve further investigation.

These results reveal that microglia modulate brain wiring at various developmental steps, starting from embryonic, post-natal and adult stages. Moreover, they underlie the importance of spatial and temporal positioning of these cells to accomplish their roles as modulators of dopaminergic axonal outgrowth, CC development and neocortical interneuron laminar distribution, which are major events in forebrain wiring.

## Conclusions and perspectives

While the concept of guidepost cell has substantially changed since its first description, including a potential motility as well as a diverse cellular identity (neuronal or glial), there are still some conserved properties: they are usually early born, immature, located at a crucial point along a pathway and able to act at short-range or by direct cell-cell contact on its target. Along these lines, recent studies have revealed that microglia cells may be to some extent, envisaged as novel guideposts during embryonic forebrain wiring. By their transient specific localization during embryogenesis they may act on restricted neuronal subgroups and modulate forebrain wiring. If a comprehensive knowledge of all microglia functions is still fragmentary, the tremendous potential of these cells in shaping and remodeling circuits, during normal and pathological conditions, opens a novel framework for our understanding of brain wiring.

## Conflict of interest statement

The authors declare that the research was conducted in the absence of any commercial or financial relationships that could be construed as a potential conflict of interest.
